# Nitrogen-Fixing Bacteria Associated with *Peltigera* Cyanolichens and *Cladonia* Chlorolichens

**DOI:** 10.3390/molecules23123077

**Published:** 2018-11-25

**Authors:** Katerin Almendras, Jaime García, Margarita Carú, Julieta Orlando

**Affiliations:** Laboratory of Microbial Ecology, Department of Ecological Sciences, Faculty of Sciences, Universidad de Chile, Santiago 7800003, Chile; katalmendras@gmail.com (K.A.); jaime.garcias@usach.cl (J.G.); mcaru@uchile.cl (M.C.)

**Keywords:** bacterial community structure, Chile, chlorolichens, Coyhaique National Reserve, cyanolichens, *nif*H gene, *Nothofagus* forest, terricolous lichens

## Abstract

Lichens have been extensively studied and described; however, recent evidence suggests that members of the bacterial community associated with them could contribute new functions to the symbiotic interaction. In this work, we compare the nitrogen-fixing guild associated with bipartite terricolous lichens with different types of photobiont: *Peltigera* cyanolichens and *Cladonia* chlorolichens. Since cyanobacteria contribute nitrogen to the symbiosis, we propose that chlorolichens have more diverse bacteria with the ability to fix nitrogen compared to cyanolichens. In addition, since part of these bacteria could be recruited from the substrate where lichens grow, we propose that thalli and substrates share some bacteria in common. The structure of the nitrogen-fixing guild in the lichen and substrate bacterial communities of both lichens was determined by terminal restriction fragment length polymorphism (TRFLP) of the *nif*H gene. Multivariate analyses showed that the nitrogen-fixing bacteria associated with both types of lichen were distinguishable from those present in their substrates. Likewise, the structure of the nitrogen-fixing bacteria present in the cyanolichens was different from that of chlorolichens. Finally, the diversity of this bacterial guild calculated using the Shannon index confirms the hypothesis that chlorolichens have a higher diversity of nitrogen-fixing bacteria than cyanolichens.

## 1. Introduction

Lichens are defined as mutualistic symbioses between a fungus (mycobiont) and a population of photosynthetic partners (photobiont). These associations can be bipartite, in which the mycobiont is associated with a green algae (chlorolichens) or a cyanobacteria (cyanolichens); and tripartite, where the three organisms are present [1]. Lichens are pioneers in the colonization of diverse environments and can develop on a diversity of substrates, such as soil, stones, or living as epiphytic organisms on plants, among others [2].

Lichens are considered one of the most successful life forms, in which the functions of each member of the association are well known: the mycobiont provides the photobiont with refuge and protection against erosion and desiccation, while the photobiont contributes with the production of organic matter through photosynthesis and, if it is a cyanobacterium, with the fixation of atmospheric nitrogen [1]. In tripartite associations, there is a separation in the functions of the photobionts, where green alga fix carbon and the cyanobacteria fix nitrogen. In bipartite cyanolichens, the cyanobacteria perform both processes, but in bipartite chlorolichens, the green algae are not able to fix nitrogen and only contributes with products of photosynthesis [3,4,5]. Therefore, it is proposed that bacteria associated with chlorolichen thalli could supply nitrogen to the association through the guild of nitrogen fixers and thus sustain part of the lichen nutrition [6].

The study of bacterial communities associated with lichen thalli is recent, although the presence of these communities has been known for a long time by determinations based on phenotypic and physiological characteristics [7,8,9,10]. These bacterial communities form a highly structured biofilm on the thallus and the possible roles that they could be carrying out in the lichen symbiosis have been described lately by metaproteomics, in vitro antagonistic activity and macromolecular hydrolytic activity. Some contributions of the bacteria associated with lichens include: nutrient supply (nitrogen, phosphorus and sulfur), resistance against biotic and abiotic factors, and support for photosynthesis through the supply of vitamin B12 [6,11,12,13,14]. On the other hand, little is known about how lichens acquire, structure, and transmit their associated bacterial communities. Lichens may carry part of their bacterial community with them when they reproduce vegetatively and propagate to a new site [15,16] although it cannot be ruled out that part of these bacteria could also be recruited from the substrate where lichens grow [17,18,19]. In fact, the communities of the lichen thalli are different from those of the substrates where they grow. Although, they share certain bacteria in common [17,20]. Moreover, the lichen substrate has been proposed as one of the sources of cyanobionts [21], reinforcing the proposal that there could be a selection of members of the symbiosis from this underlying environment [17,19,20].

The release of nitrogen compounds by diazotrophs could be particularly important for bipartite chlorolichens, since they have a non-cyanobacterial photobiont and therefore depend on nitrogen compounds from the substrate or external sources. The biological fixation of nitrogen is carried out by the nitrogen-fixing bacteria and corresponds to the reduction of molecular nitrogen (N_2_) to biologically available ammonium (NH_4_^+^), catalyzed by the nitrogenase enzyme which is encoded in the *nif*HDK operon [22]. These bacteria could live on or within the lichen thallus or in the substrate influenced by the mycobiont hyphae [6].

In this study, we analyze the diversity of the guild of nitrogen-fixing bacteria associated with terricolous lichens (i.e., lichens using soil as the substrate) with different types of photobiont: bipartite chlorolichens of the genus *Cladonia* and bipartite cyanolichens of the genus *Peltigera*. We hypothesized that (i) the bacterial communities from the thalli and the substrates are different but share some members and (ii) bipartite chlorolichens have a higher diversity of nitrogen-fixing bacteria than bipartite cyanolichens. For this, the diversity of the diazotrophic guilds was assessed by a culture-independent fingerprinting approach targeting a molecular marker for nitrogen fixation (*nif*H gene coding nitrogen reductase).

## 2. Results

### 2.1. Molecular Identification of Mycobionts

Molecular identification of lichen mycobionts by the analysis of their ribosomal markers showed that, in the 25 *Peltigera* samples there was a total of six mycobiont haplotypes (PM1, PM2, PM4, PM5, PM6, and PM8), whose names correspond to those in Zúñiga et al. [23], and in the 25 *Cladonia* samples there was a total of 11 mycobiont haplotypes (CM1, CM2, CM3, CM4, CM5, CM6, CM7, CM8, CM9, CM10, and CM11).

**Figure A1:**
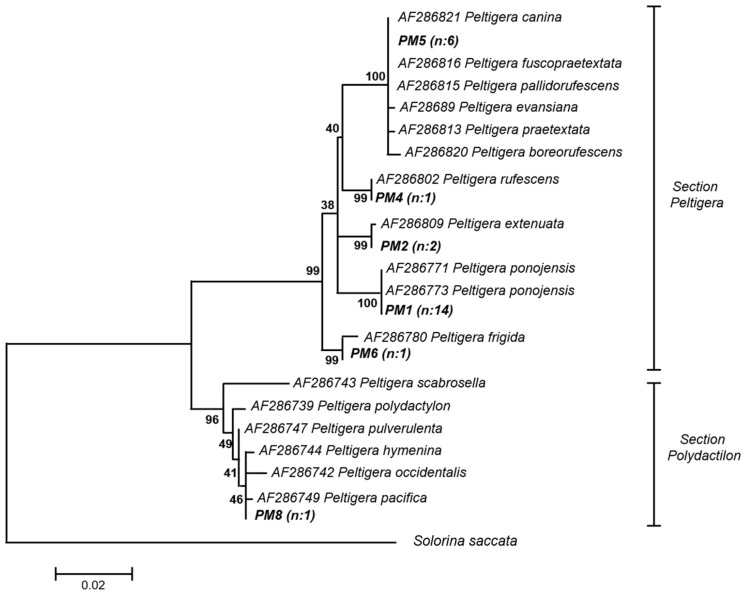
Phylogenetic tree of *Peltigera* operational taxonomic units (OTUs) by Bayesian inference. Bootstrap support values are indicated at the nodes and the bar indicates the genetic distance. The mycobionts from this study, named as PM followed by a number, are shown in bold. The number of specimens per haplotype is shown in parentheses. The division of the species into sections corresponds to Zúñiga et al. [23]. *Solorina saccata* (accession number DQ973044) sequence was used as outgroup.

The identification of *Peltigera* haplotypes was carried out by phylogenetic analysis of Bayesian inference with 18 *Peltigera* 28S rRNA sequences downloaded from GenBank, see Figure A1. According to this, PM1 was related to *P. ponojensis*, PM2 to *P. extenuata*, PM4 to *P. rufescens*, PM5 to the *P. canina* lineage (*P. evansiana*, *P. canina*, *P. “fuscopraetextata”*, *P. “pallidorufescens”*, *P. praetextata*, and *P. “boreorufescens”*), PM6 to *P. frigida* and PM8 to the *P. hymenina* lineage (*P. polydactylon*, *P. occidentalis*, *P. scabrosella*, *P. pacifica*, *P. hymenina, P. truculenta*, and *P. pulverulenta*). PM5 and PM8, according to recent phylogeny analyses and distribution patterns most-likely correspond to *P. “fuscopraetextata”* and *P. truculenta*, respectively [19,24,25].

**Figure A2:**
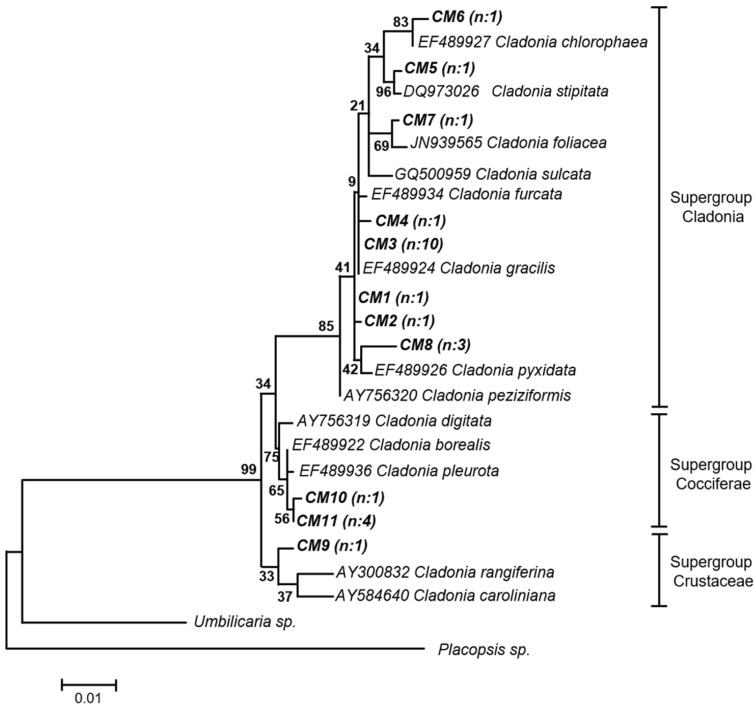
Phylogenetic tree of *Cladonia* operational taxonomic units (OTUs) by Bayesian inference. Bootstrap support values are indicated at the nodes and the bar indicates the genetic distance. The mycobionts from this study, named as CM followed by a number, are shown in bold. The number of specimens per haplotype is shown in parentheses. The division of the species into supergroups corresponds to Stenroos et al. [73]. *Umbilicaria* sp. (accession number FN186084) and *Placopsis* sp. (accession number AY212845) sequences were used as outgroups.

Similarly, for the identification of *Cladonia* haplotypes, a phylogenetic analysis of Bayesian inference with 13 *Cladonia* 28S rRNA sequences downloaded from GenBank was carried out, see Figure A2. The haplotype CM5 was robustly related to *C. stipitata* and CM6 to *C. chlorophaea*; also, CM7 was related to *C. foliacea* and CM8 to *C. pyxidata*, although with not very good support. The rest of the haplotypes exhibited low bootstrap support, so they were classified in supergroups. CM1, CM2, CM3, and CM4 were grouped within the Cladonia supergroup, which also includes the species mentioned above; CM9 within the Crustaceae supergroup; and CM10 and CM11 within the Cocciferae supergroup.

### 2.2. Genetic Structure of Nitrogen-Fixing Bacteria Associated with Peltigera and Cladonia

The genetic structure of the nitrogen-fixing guild associated with both *Peltigera* and *Cladonia* was determined by terminal restriction fragment length polymorphism (TRFLP) with two different restriction enzymes (*Hae*III and *Hha*I). For this, the *nif*H gene was amplified from substrates and thalli of both lichens. Six samples of *Cladonia* thallus (C2, C3, C16, C19, C21, and C22) did not show amplification of the *nif*H gene, despite several attempts to optimize the amplification. Therefore, these samples were discarded from the following analyses.

**Figure A3:**
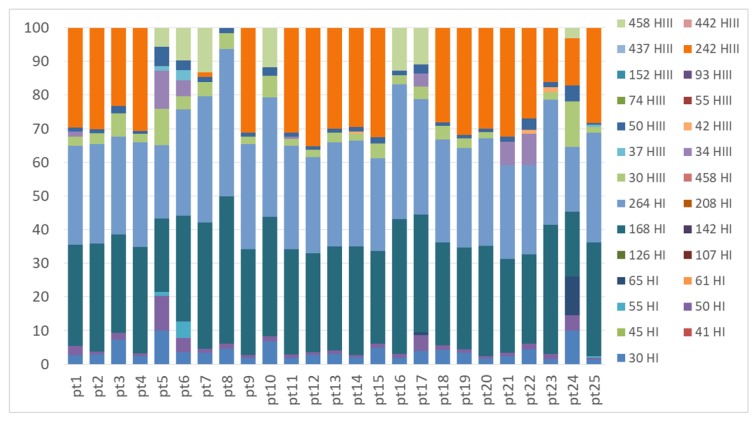
TRFLP profiles of the *nif*H gene obtained from *Peltigera* thallus samples with the restriction enzymes *HhaI* (TRFs indicated in the legend with a number followed by HI) and *HaeIII* (TRFs indicated in the legend with a number followed by HIII).

**Figure A4:**
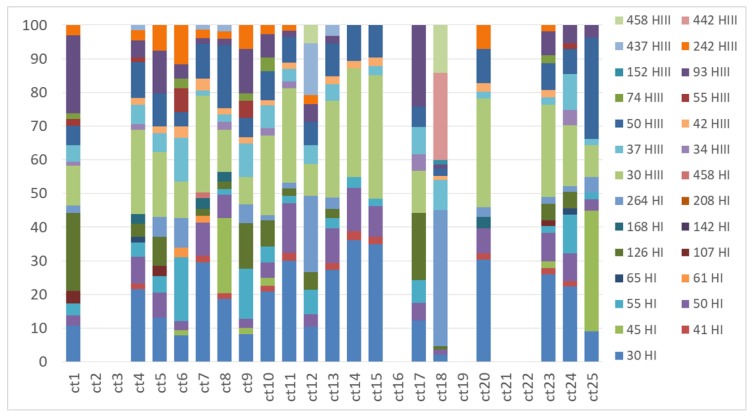
TRFLP profiles of the *nif*H gene obtained from *Cladonia* thallus samples with the restriction enzymes *HhaI* (TRFs indicated in the legend with a number followed by HI) and *HaeIII* (TRFs indicated in the legend with a number followed by HIII).

**Figure A5:**
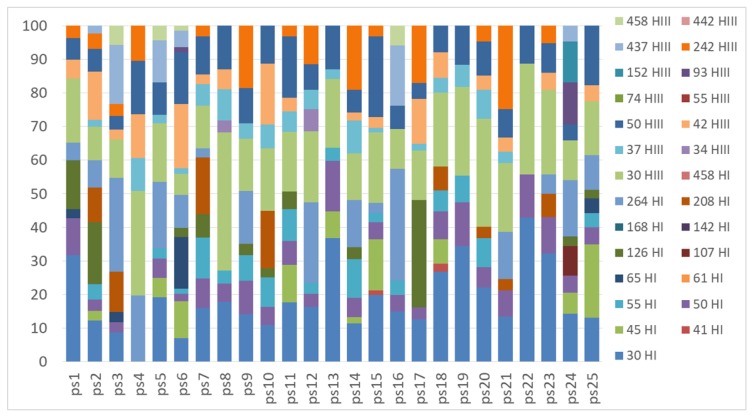
TRFLP profiles of the *nif*H gene obtained from the substrate samples underlying *Peltigera* lichens with the restriction enzymes *HhaI* (TRFs indicated in the legend with a number followed by HI) and *HaeIII* (TRFs indicated in the legend with a number followed by HIII).

**Figure A6:**
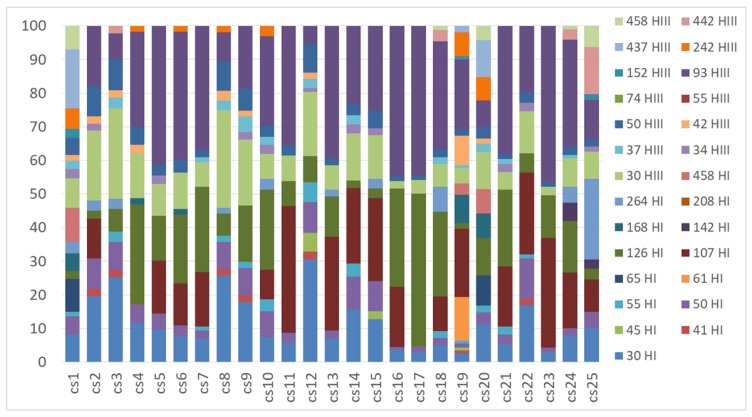
TRFLP profiles of the *nif*H gene obtained from the substrate samples underlying *Cladonia* lichens with the restriction enzymes *HhaI* (TRFs indicated in the legend with a number followed by HI) and *HaeIII* (TRFs indicated in the legend with a number followed by HIII).

From *Peltigera* and *Cladonia* thalli, 13 and 26 terminal restriction fragments (TRFs) were obtained, respectively, considering both enzymes, see Figure A3 and Figure A4. Of these, 13 were present in both lichens, and only *Cladonia* lichens show exclusive TRFs. On the other hand, from the *Peltigera* and *Cladonia* substrates, 21 and 24 TRFs were obtained, respectively, see Figure A5 and Figure A6, and more of them were present in both substrates. In addition, 13 TRFs were found in both *Peltigera* thalli and their substrates, while 23 TRFs were present in both *Cladonia* thalli and their substrates, although in both cases the abundance of each TRF was different in thalli and substrates.

A tentative identification of TRFs with bacterial groups reported in a comprehensive database of *nif*H genes was performed by comparing experimental and predicted TRFs. A possible allocation of a TRF was recorded when the experimental and the predicted TRFs were consistent for both enzymes. In addition, the environment in which the probable microorganism has been found was checked. In general, TRFs could not be assigned to a single species, but rather to a group of related species. Although, in some cases, a fragment could also be associated with unrelated species, see Table 1. Using this criterion, alphaproteobacteria and cyanobacteria (*Nostocales*) were found in the whole set of samples. The highest amount of TRFs was identified in profiles derived from *Cladonia* substrates, where TRFs were associated with *Alphaproteobacteria* (*Rhizobiales* and *Rhodobacterales*), *Betaproteobacteria* (*Burkholderiales*), *Gammaproteobacteria* (*Pseudomonadales*), *Actinobacteria* (*Frankiales*), *Firmicutes* (*Clostridiales*), *Cyanobacteria* (*Nostocales*), and uncultured microorganisms. Conversely, only four TRFs were identified in the *Peltigera* substrates.

**Figure 1:**
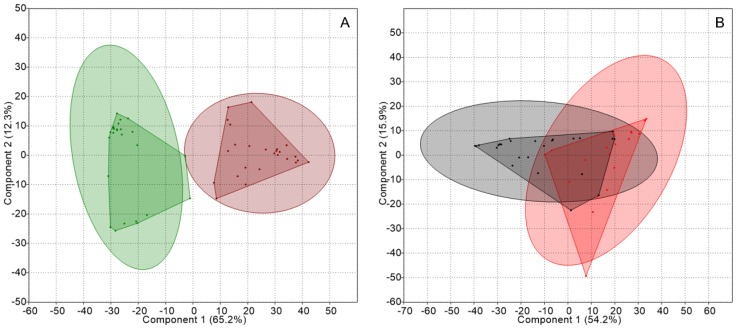
Principal component analyses (PCA) of the genetic structure of the nitrogen-fixing guild associated with thalli and substrates from each lichen genus: (**A**) *Peltigera* thalli (green) and substrates (brown), and (**B**) *Cladonia* thalli (red) and substrates (gray). Concentration ellipses (ovals surrounding the convex hulls) estimate the region where 95% of population points are expected to fall.

Firstly, the profiles were analyzed to compare thalli and substrates from each lichen genus. Principal component analyses (PCA) of TRFLP data from *Peltigera* thalli and substrates formed separated groups, as shown in Figure 1, and showed significant differences according to an analysis of similarities (ANOSIM); while an analysis of similarity percentage (SIMPER) gave a dissimilarity of 75.6%, see Table 2. In contrast, an overlap was observed between samples from *Cladonia* thalli and substrates, with a dissimilarity of 59.2%.

**Figure 2:**
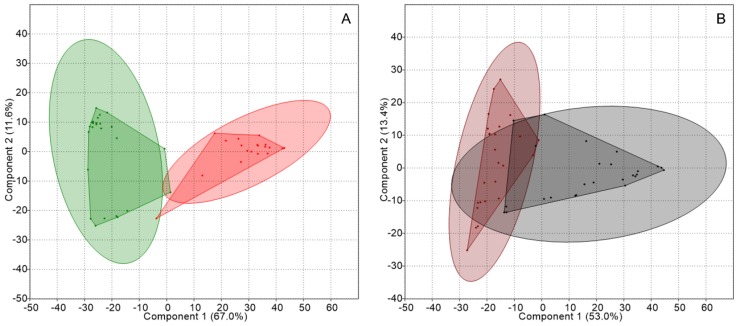
PCA of the genetic structure of the nitrogen-fixing guild associated with thalli and substrates: (**A**) *Peltigera* thalli (green) and *Cladonia* thalli (red), and (**B**) *Peltigera* substrates (brown) and *Cladonia* substrates (gray). Concentration ellipses (ovals surrounding the convex hulls) estimate the region where 95% of population points are expected to fall.

Subsequently, data were grouped according to the micro-habitat occupied by the bacterial guild (thallus or substrate). PCA obtained by comparing the TRFLP data from the thalli and from the substrates of both kinds of lichens, showed that, in the former, two well-defined groups existed according to the lichen type, see Figure 2, which were significantly different according to ANOSIM and showed a dissimilarity of 80.7%, as shown in Table 2. In the case of the substrates, they formed two separate groups according to the lichen identity but exhibited an overlapping area.

**Figure 3:**
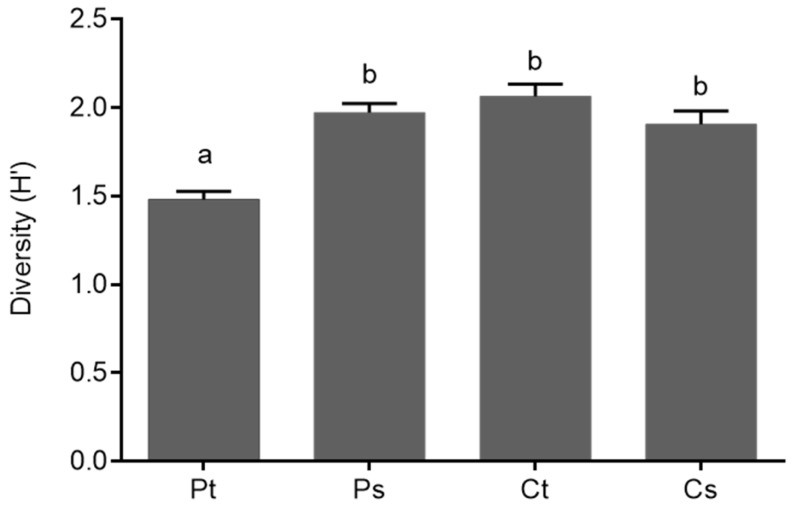
Diversity of nitrogen-fixing bacteria associated with thalli and substrates of *Peltigera* and *Cladonia* lichens. Means and standard error are shown. Different letters above the bars represent significant differences (ANOVA-Tukey; *p* ≤ 0.05). Pt: *Peltigera* thalli; Ps: *Peltigera* substrates; Ct: *Cladonia* thalli; Cs: *Cladonia* substrates.

Finally, the Shannon diversity index (H’) values of the nitrogen-fixing bacteria from thalli and substrates of both types of lichens were compared using an analysis of variance (ANOVA-Tukey, *p* ≤ 0.05). A higher diversity of TRFs was found for the nitrogen-fixing bacteria associated with the *Cladonia* thalli than the *Peltigera* thalli, whilst the diversity of nitrogen-fixers from *Peltigera* substrates and *Cladonia* substrates was similar, as shown in Figure 3.

## 3. Discussion

The identification of lichens, at least at the genus level, is essential to test the hypothesis of this work due to the comparison between two bipartite lichens with different photobionts; chlorolichens of the genus *Cladonia*, where the photobiont contributes to the fixation of carbon, and cyanolichens of the genus *Peltigera*, where the photobiont contributes to the fixation of carbon and nitrogen. Although the *Peltigera* genus includes tripartite associations involving the fungus, a green alga (*Coccomyxa*), and a cyanobacterium (*Nostoc*) [27], in Chile only bipartite species have been reported [23,28,29,30]. The identification based on morphological aspects could be difficult due to the existence of cryptic species [31]. Therefore, the identification of the mycobionts in this work was confirmed using the 28S rRNA gene of fungi as the molecular marker, which is a conserved gene with taxonomic and phylogenetic value [32]. Through phylogenetic analyses, we were able to distinguish six haplotypes of mycobionts for *Peltigera* and 11 for *Cladonia*, all related to bipartite species. There are few studies on the lichen diversity of the *Peltigera* genus in the Aysén region (Chile) [17,19,23,29], but all *Peltigera* species identified in this work were previously reported for the Coyhaique National Reserve [17,19,23]. On the other hand, some of our *Cladonia* haplotypes were highly related to species of this genus, including *C. stipitata* (CM5) and *C. chlorophaea* (CM6). Quilhot et al. [29] made a compilation of lichen species, including *Cladonia*, present in the Aysén region, in which the presence of *C. stipitata* was not registered. Although, this genus appears to be highly represented in the region. This species was first described in North America [33] and, to date, there are no records of this species in Chile or South America. This demonstrates that although the south of Chile has a great diversity of lichens, it is still an insufficiently explored area [17,23].

Nitrogen-fixing bacteria are of great ecological importance because they provide the main natural biological source of nitrogen in the biosphere fixed from the atmosphere, as opposed to the industrial fixation carried out by the Haber–Bosch process [34]. These bacteria are diverse and most have not been cultivated [35]. For this reason, it is essential to use molecular markers for research in which the phylogeny, diversity, and abundance of nitrogen-fixing microorganisms are studied. Nitrogen fixation is carried out by the enzyme nitrogenase, whose multiple subunits are encoded by the *nif*H, *nif*D, and *nif*K genes [36]. Of those, the *nif*H gene is the most represented in the databases [11,37] and is a highly conserved gene [38], so it is an adequate molecular marker for the analysis of the diversity of nitrogen-fixing organisms.

The genetic structure of the nitrogen fixers present in the thalli compared to those in the substrates was significantly different for both lichens. Although, *Peltigera* had a higher dissimilarity (75.1%) than *Cladonia* (59.1%). It has been proposed that, when vegetative lichen propagules are dispersed, nitrogen fixers are uploaded from the new location so they are better adapted to the new environmental conditions [15,39]. Therefore, the lower dissimilarity of *Cladonia* thalli and substrates suggests that these lichens could be less selective in recruiting nitrogen-fixing bacteria from the substrate to increase the diversity of this guild on their thalli, which could compensate for the inability of the algal photobiont to fix nitrogen. The influence of photobionts on bacterial communities related to lichen thalli was described previously by Hodkinson et al. [15], who found a predominance of *Alphaproteobacteria*, mainly *Rhizobiales*, and argued that the differences in bacterial community composition associated with lichen thalli could be ascribed to the availability of fixed nitrogen. Our results from an in silico TRFLP analysis showed differences among the putative identified diazotrophs, suggesting that the photobiont type (i.e., cyanobacterial or green-algal symbiont) could affect the structure of the guild. In fact, while in *Peltigera* thalli we mainly identified *Cyanobacteria* and *Alphaproteobacteria*, in *Cladonia* thalli several bacterial groups appeared, such as *Alphaproteobacteria*, *Actinobacteria*, *Firmicutes*, and *Cyanobacteria*. Similar differences were observed when analyzing the substrates from both lichen types, the guild from *Cladonia* substrates being the most diverse, which includes the aforementioned bacterial groups in addition to *Betaproteobacteria*, *Gammaproteobacteria*, and some TRFs related to uncultured microorganisms. In previous studies, similar nitrogen-fixing guilds for *Cladonia* chlorolichens were reported, with a predominance of *Alphaproteobacteria* and less abundance of *Actinobacteria* and *Betaproteobacteria* [40]. Recently, a metagenomic analysis of the *Peltigera ponojensis* microbiome showed a predominance of the class *Alphaproteobacteria*, with *Rhizobiales*, *Sphingomonadales*, and *Rhodospirillales* being the most important orders [41].

The presence of *Alphaproteobacteria* appears to be common in the microbiota of the thallus and substrate of lichens [6,39,40,41,42,43,44], with a predominance of *Rhizobiales*, some of which are well-known symbiotic nitrogen fixers. Among them, a lichen-associated lineage (designated as LAR1) has been described. However, its capacity to fix nitrogen has not been demonstrated [15,37,43]. In addition, in a metaproteomic analysis of the bacterial communities associated with *Lobaria pulmonaria*, although the *Rhizobiales* comprises several nitrogen-fixers, the nitrogen fixation could not be assigned to this group [45]. Regardless of this, *Rhizobiales* can contribute to other functions to the lichen symbiosis, such as in the biosynthesis of phytohormones and vitamins [13,44]. On the other hand, free-living nitrogen-fixers associated with the lichen thallus have also been described which belong to unrelated lineages such as *Actinobacteria* of the genus *Frankia* [43,46], *Firmicutes* from the genera *Bacillus* and *Clostridium* [11,43], *Rhodobacterales* [41,47], *Burkholderiales* [11,39,47], and *Pseudomonadales* [11,47]. Therefore, the higher diversity of free-living nitrogen-fixers (non-rhizobial bacteria), such as those found in the *Cladonia* thalli, could account for a greater contribution of nitrogen to chlorolichens than rhizobial bacteria. Finally, in addition to the main cyanobiont, other cyanobacteria have been reported as part of the microbiota of lichen thalli and substrates [21,41], which as free-living nitrogen-fixers, can also contribute with part of the nitrogen input to the symbiosis.

The differences observed in the nitrogen-fixing guild from both lichen types could also be explained by the different growth forms of the two lichen genera, as *Peltigera* is a foliose species whilst *Cladonia* is fruticose. Foliose lichens usually come into contact with the underlying substrate over a wider surface compared to fruticose lichens. It is probable that the difference in contact with their substrates affects the selection of bacteria that are associated with the thalli, which is performed through the production of metabolites and certain enzymatic activities [17,48] with antibiotic, cytotoxic, and antiviral effects [49,50,51,52,53,54]. These micro-environmental modifications (i.e., niche construction) could already shape the species interactions [55]. In fact, the structure of the lichen bacterial microbiota is influenced by several factors, including intrinsic lichen factors such as mycobiont identity and metabolite diversity [6,17,43], photobiont identity or lichen growth type [15], and extrinsic factors like biogeography and environmental context [15,20,40,56,57]. Therefore, we cannot rule out that other factors, besides the presence or absence of the ability to fix nitrogen from the photobionts, could shape the nitrogen-fixing bacterial guilds associated with lichens. Taken together, the evidence suggests that both intrinsic and extrinsic factors may be selecting specific guilds, as is the case of nitrogen fixers, which are necessary for the development of a healthy thallus.

The richness and diversity of nitrogen-fixing bacteria in *Cladonia* thalli was greater than in *Peltigera* thalli. *Cladonia* lichens possess green alga as the photobiont which, unlike *Peltigera* cyanobionts, do not possess the ability to fix nitrogen [15]. Therefore, it is expected that this more diverse guild in *Cladonia* lichens compared to in *Peltigera* lichens, has the purpose of supplying the necessary nitrogen for their development [8,58,59], although activity assays are required to confirm this assumption. In any case, this has little relation with the capacity of nitrogen fixation in *Cladonia*, since nitrogen fixation is regulated differently in cyanobacteria and proteobacteria (the most abundant group of bacteria communities found associated with lichens). Generally, proteobacteria fix nitrogen at specific times and when micro-climatic conditions are adequate, while cyanobacteria designate specific cells as nitrogen fixers [4].

Finally, as was observed in the present work and elsewhere [14], there is a specific bacterial community associated with the lichen thallus, which differs from the bacterial community present in the underlying substrate, that fulfills particular functions in the association and confirms the notion that lichens consist of multi-species symbioses. Through multiomic approaches, there is evidence of a functional contribution of the lichen-associated bacteria to the complete meta-organism [46], such is the case of the guilds of nitrogen-fixing bacteria present in both *Cladonia* and *Peltigera* lichens.

## 4. Materials and Methods

### 4.1. Study Site and Sampling

We collected 25 *Peltigera* and 25 *Cladonia* thalli (n = 50 lichen samples) and their associated substrate (i.e., soil; n = 50 substrate samples) from a 2 Ha plot of a secondary forest of *Nothofagus pumilio* in the Coyhaique National Reserve (Aysén Region, Chile; 45°31′42.96′′ S, 72°1′51.95′′ W, 800 m.a.s.l.) which was originated after anthropogenic fires in the middle of the 20th century. The soil is derived from aeolian volcanic ash deposits and the annual precipitation is approximately 1350 mm [60]. These two genera of lichens were selected because both are terricolous (they share the soil as substrate) and coexist in the same habitat, which should reduce the variability between the samples by extrinsic factors. In addition, they are bipartite lichens that have a single photobiont (cyanobiont and chlorobiont, respectively). Both genera are abundant and diverse in the study site, which allowed us to collect a representative sample of the area with several biological replicas [23,29].

The samples were placed in paper bags to avoid deterioration and decrease humidity and transported in cooler containers at low temperature. In the laboratory, the lichen samples were stored in paper bags at room temperature with low humidity, while the substrate samples were sieved and stored in plastic tubes at 4 °C.

### 4.2. Pre-Treatment of Samples and DNA Extraction

The lichen thalli were superficially cleaned with a sterile brush and spatula to remove adhered substrate residues. Subsequently, the samples were washed with sterile distilled water and air dried at room temperature.

DNA extraction from 100 mg of lichen thalli (mechanically fractioned) and 100 mg of substrates was carried out using the PowerSoil DNA Isolation kit (Qiagen Laboratories Inc., Venlo, Netherlands) according to the manufacturer’s instructions. Quality and integrity of the extracted DNA were visualized electrophoretically in 0.8% (*w*/*v*) agarose gels in TAE 1X buffer (40 mM Tris-acetate, 1 mM EDTA [pH 8.0]) stained with GelRed (Biotium, Fremont, CA, USA). All DNA samples were stored in TE buffer (10 mM Tris-HCl and 1 mM EDTA [pH 8.0]) at −20 °C.

### 4.3. Molecular Identification of Mycobionts

The molecular identification of the mycobionts of *Peltigera* and *Cladonia* lichens was performed by PCR amplification of the fungal 28S rRNA gene (LIC24R and LR7 primers) [27]. PCR mixes were prepared using GoTaq^®^ Green Master Mix (GoTaq DNA polymerase in Green GoTaq Reaction Buffer [pH 8.5], 200 μM of each dNTP and 1.5 mM MgCl_2_) (Promega, Madison, WI, USA) and amplified in a Maxygene II thermocycler (Axygen, Tewksbury, MA, USA). Cycling conditions consisted of an initial denaturation step at 94 °C for 1 min; then 30 cycles of 94 °C for 30 s, 52 °C for 30 s, and 72 °C for 1 min 30 s; and finally an extension of 72 °C for 4 min. The quality and size of the amplicons were determined electrophoretically as described previously, except that 1.2% (*w*/*v*) agarose gels were used.

All amplicons were sequenced with the forward primers in the Genetic Analyzer 3730XL (Applied Biosystems, Foster City, CA, USA) using a sequencing service (Macrogen, Seoul, Korea). DNA sequences were visually checked and manually edited on Mega 5.0 software [61] and aligned with the Muscle alignment tool [62]. Then, they were grouped in operational taxonomic units (OTUs) according to criteria of 100% identity; therefore, each OTU corresponds to a different haplotype. The sequences obtained were deposited in the GenBank database under accession numbers KF718515 to KF718524, KF718527 to KF718529, KF718535 to KF718538, KF718541, KF718543 to KF718547, KF718556 to KF718557 (*Peltigera* lichens), and MH998088 to MH998112 (*Cladonia* lichens).

Using one representative for each OTU, phylogenetic analyses of Bayesian inference (5,000,000 generations) were carried out in the MrBayes v3.2.2 program [63]. The best nucleotide substitution model was determined by the jModelTest v2.1.3 program [64]. In addition, support at the nodes was calculated with the bootstrap method [65]. The graphic representation of the trees was obtained with the program FigTree v1.4.0. Only the sequences deposited in the NCBI GenBank nucleotide database [66] of the species closest to each of the OTUs were considered to create the phylogenetic trees.

### 4.4. Terminal Restriction Fragment Length Polymorphism

The genetic structure of the nitrogen-fixing guild associated with both *Peltigera* and *Cladonia* lichen thalli and substrates was determined by obtaining terminal restriction fragment length polymorphisms (TRFLP). To do so, the bacterial *nif*H gene, which encodes the nitrogenase-reductase subunit, was amplified from the isolated DNA of lichen thalli and their corresponding substrates, with the forward primer nifHF, labeled with 6-FAM (6-carboxyfluorescein), and the reverse primer, nifHR [67]. PCR mixes, as well as the size and quality control of the amplicons, were carried out as mentioned earlier. Cycling conditions consisted of an initial denaturation step at 94 °C for 1 min; then 40 cycles of 94 °C for 30 s, 52 °C for 30 s and 72 °C for 30 s; and an extension at 72 °C for 7 min.

Amplicons were purified using the PCR Clean-Up System kit (Promega, Madison, WI, USA), and were independently incubated for 16 h at 37 °C with two different restriction enzymes: *Hae*III and *Hha*I (Fermentas, Waltham, MA, USA) according to the manufacturer´s instructions. Subsequently, an alcohol DNA precipitation was carried out and the products were resuspended in TE buffer. The fragments were analyzed by capillary electrophoresis on an automated Genetic Analyzer ABI3730XL (Applied Biosystems, Foster City, CA, USA) using a sequencing service (Macrogen, Seoul, Korea). The size, height, and area of the terminal restriction fragments (TRFs) were determined by comparison with the GeneMapper software v3.7 (Applied Biosystems, Foster City, CA, USA).

The signals in the profiles were edited as described by Almasia et al. [68]. Briefly, in order to discard peaks corresponding to PCR primers, only fragments of 30 bp or longer were considered in the data analysis. Patterns from different samples were normalized to homogenize the total fluorescence units by an iterative standardization procedure [69]. Additionally, profiles were manually aligned to avoid erroneous identification of TRFs by the expected shift in fragment sizes due to electrophoresis. Finally, the relative abundance of each TRF, as a percentage, was determined by calculating the ratio between the height of the peak and the normalized total peak height of each sample.

Additionally, to eliminate TRFs associated with the cyanobiont *Nostoc*, the *nif*H gene sequence of *Nostoc flagelliforme* (accession number KU886163.1) was analyzed in silico with the software BioEdit [70] to determine the position of cleavage sites of *Hae*III and *Hha*I enzymes from the hybridization site of the nifHF primer.

### 4.5. Data Analyses

For the assignment of TRFs to a bacterial group, an in silico TRFLP analysis was carried out with the TRiFLe Java-based program [26] using a database containing 32,954 aligned nitrogenase *nifH* sequences (http://www.css.cornell.edu/faculty/buckley/nifh.htm) [38]. The experimental values were corrected using the correction formula of Kaplan and Kitts [71].

The estimation of the genetic diversity of the nitrogen-fixing guilds was calculated using the Shannon (H’) index. On the other hand, the similarity between these bacterial guilds was determined by principal component analyses (PCA) based on the Bray–Curtis dissimilarity index. In addition, analyses of similarity (ANOSIM) and similarity percentages (SIMPER) were calculated between bacterial communities. All these analyses were performed using the PAST software v 3.11 [72].

Finally, the difference in genetic diversity values was evaluated by ANOVA and Tukey post hoc test in the software Graphpad Prism v5.01 (Graphpad Software Inc., San Diego, CA, USA).

## 5. Conclusions

Although the structure of the nitrogen-fixing guild was different between thalli and substrates of both types of lichens, thalli shared some bacteria with the substrates; therefore, the latter could be a source of nitrogen fixers to conform the lichen-associated bacteria.

On the other hand, the diversity of the nitrogen-fixing guild was lower in the *Peltigera* bipartite cyanolichens than in the *Cladonia* bipartite chlorolichens, which could be explained by the fact that cyanobionts contribute with nitrogen-fixation to the symbiosis while lichens with a green algae as the photobiont require nitrogen fixers associated with their thalli for a healthy development.

## Figures and Tables

**Table 1:** Tentative identification of the *nif*H terminal restriction fragments (TRFs) by an in silico analysis of the profiles obtained from thalli and substrates of *Peltigera* and *Cladonia* lichens.

*Hha*I	*Hind*III	Putative Identification	Pt	Ps	Ct	Cs
58 (0)	40 (3)	*Alphaproteobacteria; Rhodobacterales*				
	90 (6)	uncultured bacterium				
	154 (0)	*Actinobacteria; Frankiales*				
	465 (7)	*Cyanobacteria; Nostocales*				
144 (3)	53 (5)	*Actinobacteria; Frankiales*				
		*Alphaproteobacteria; Rhizobiales*				
		*Betaproteobacteria; Burkholderiales*				
		*Gammaproteobacteria; Pseudomonadales*				
		*Firmicutes; Clostridiales*				
		uncultured bacterium				
144 (3–6)	465 (4–10)	*Cyanobacteria; Nostocales*				
465 (4–24)	53 (3–5)	*Firmicutes; Clostridiales*				
		uncultured bacterium				

Pt: *Peltigera* thallus; Ps: *Peltigera* substrate; Ct: *Cladonia* thallus; Cs: *Cladonia* substrate. The experimental TRF length is shown for each enzyme and the drift from the predicted TRF according to TRiFLe [26] is shown in parentheses. The presence/absence of a TRF in each type of sample is indicated by a dark/empty square.

**Table 2:** Analysis of similarity of the terminal restriction fragment length polymorphism (TRFLP) profiles of the *nif*H gene obtained from thalli and substrates of *Peltigera* and *Cladonia* lichens.

Samples	ANOSIM R	ANOSIM *p*	SIMPER Dissimilarity (%)
TRFLP-Pt	0.9355	0.0001	75.6
TRFLP-Ps			
TRFLP-Ct	0.3871	0.0001	59.1
TRFLP-Cs			
TRFLP-Pt	0.9427	0.0001	80.7
TRFLP-Ct			
TRFLP-Ps	0.6212	0.0001	65.6
TRFLP-Cs			

TRFLP-Pt: *Peltigera* thallus profile; TRFLP-Ps: *Peltigera* substrate profile; TRFLP-Ct: *Cladonia* thallus profile; TRFLP-Cs: *Cladonia* substrate profile. The R and *p* values derive from ANOSIM comparisons and the dissimilarity percentages from SIMPER comparisons; both analyses were performed using the Bray–Curtis index.
